# Body image perception, eating disorder behavior, self-esteem and quality of life: a cross-sectional study among female medical students

**DOI:** 10.1186/s40337-023-00945-2

**Published:** 2023-12-15

**Authors:** Ganesh Kumar Mallaram, Pragya Sharma, Dheeraj Kattula, Swarndeep Singh, Poojitha Pavuluru

**Affiliations:** 1 Department of Psychiatry, Sri Padmavati Medical College for Women, Sri Venkateswara Institute of Medical Sciences, Tirupati, Andhra Pradesh India; 2 Clinical Psychologist, Psyche in Motion, New Delhi, India; 3 Department of Psychiatry, Christian Medical College, Vellore, Tamilnadu India; 4 Department of Psychiatry, Vardhman Mahavir Medical College & Safdarjung Hospital, Delhi, India; 5 Sri Padmavathi Medical College for Women, Sri Venkateswara Institute of Medical Sciences, Tirupati, Andhra Pradesh India

**Keywords:** Eating disorders, Body Image perception, Self-esteem, Quality of life

## Abstract

**Background:**

Eating disorders are strongly associated with body image concerns. Eating disorders tend to significantly impact the current and future health and quality of life of affected persons, their caregivers, and society. As body image is based on a social construct of ideal body image, it is essential to evaluate it in its cultural context.

**Methods:**

The current study explored the relationship among body image perception, perceived stress, eating disorder behaviour and quality of life among female medical students (n = 777). Measurements included Body Shape Questionnaire, Body Image Quality of Life Inventory, Eating Attitudes Test-26 and Rosenberg Self-Esteem Scale. Multivariate analysis was conducted.

**Results:**

There was a significant correlation between eating disorder behaviour and perceived body shape, body image, quality of life and self-esteem among our study participants. We also found eating disorder status was significantly associated with BMI, perceived body shape, quality of life and self-esteem.

**Conclusions:**

This is of clinical implication to female medical students and healthcare professionals to engage early in primary and secondary prevention of eating pathologies. Increasing awareness of these facts among female students can help identify at-risk students and help them seek timely medical help.

## Abstract (plain language summary)

Eating disorders significantly impact the current and future health and quality of life of affected persons, their caregivers, and society. Young people are persistently flooded with social media conceptualizations of what beauty should look like. The current study explored the relationship between how we perceive our body, perceived stress, maladaptive eating behaviours and quality of life among female medical students (n=777). Measurements included those measuring perceived body shape and body image, quality of life, eating attitudes, and self-esteem. We found that a preoccupation with weight and food, crash diets, fasting, binge eating, and purging behaviours was related to how we perceived our body shape, our quality of life and self-esteem among the study participants. This is important for female medical students and healthcare professionals because it enables them to identify students at risk of eating disorders and assist them in obtaining timely medical help, thus promoting early prevention.

## Introduction

Eating disorders are regarded to be severe mental health illnesses that have significant adverse consequences for health as well as quality of life. Several eating disorders have been strongly related to concerns regarding body image. Abnormal eating behaviours and dysfunctional body image are essential for the development of various eating disorders [1].

Body image is the intuitive portrayal a person holds of their body, regardless of their appearance. *Body image* is, thus, a complex concept including self-observations, cognitions, emotions, memories, fantasies as well as behaviors related to an individual’s body, both conscious and unconscious.

Body image is how an individual considers or imagines their body and how it appears to others. Gender and social standards might influence the ideal physique. A person has a poor body image when they are preoccupied with the fictitious flaws of their physical appearance [2].

Body image misperception is commonly seen among people and is an important component of various mental illnesses like obsessive–compulsive and related disorders and eating disorders. An Indian study shows a multitude of females misperceive how much they weigh [3]. This weight misperception could result in concerns regarding body image, eating disorders, as well as harmful eating habits [4].

An inconsistency between the perception of body image and its idealized image can lead to body dissatisfaction [5]. Body image distortions are uncomfortable and can have disastrous results. Poor body image may affect, both, physical and psychological health and impact self-esteem, mood, and social and occupational functioning [5].

A significant discrepancy exists between the socially accepted body shape and the actual body of adolescents and young adults. Young people are persistently flooded with social media conceptualizations of what beauty should look like, including the glorification of unrealistic body shapes and types. Positive body image comprises appreciation of and being able to embrace their body [6]. In contrast a negative body image relies on a pessimistic personal appraisal of one’s body [7].

Eating disorder behaviour can be defined as pathological eating attitudes and habits (like binging and purging). Disordered eating research has been usually carried out with adolescents in high-income Western countries [8]. While it was believed that Asian countries were less vulnerable to body image concerns, recent evidence suggests that the prevalence of body image disorders in Asia is becoming comparable to that in Europe and America [9].

Eating disorders tend to significantly impact the current as well as subsequent health and life quality of the afflicted, their caregivers, and society. While there have been limited studies on the same in India, individuals suffering from eating disorders exhibited substantially poorer life quality than non-eating disorder controls, according to a US general population cohort research [10].

Many people view medical school as challenging, and medical students report a high prevalence of psychological morbidity, including stress, interpersonal problems, suicide ideation, and mental disorders [11]. Medical students, like all students, experience academic and personal pressures. However, the medical field may add distinct stressors, including long hours, high expectations, and demanding clinical rotations. These factors can exacerbate body image concerns, making it essential to understand their specific experiences [12]. It is, thus, expected that the second greatest attention among faculty in medical institutions in India, (after medical education) has been given to the mental health of medical students [13].

Medical students are more susceptible to mental health challenges due to the rigorous nature of their studies and the high expectations placed upon them [14]. Medical training constitutes a complete and dedicated obligation for students, involving academic obligations, engagements, interpersonal behavior, patient care, and support. The mental well-being of medical students is consistently influenced during their education, attributable to extended study and work periods, substantial curriculum load, tests, rivalry with peers, uninspiring surroundings, inadequate sleep, feelings of isolation, and various other elements that disrupt their daily personal, social, and familial routines [15]. Addressing body image concerns early can prevent the development of more severe mental health issues in the future.

Research conducted in India indicates that body image worries are observed in around 10% to 30% of adolescent and college-going females [16, 17]. Nevertheless, a separate study in India focusing on medical and paramedical students (including both genders, with a total of 454 participants) found that 41% to 48% of them engage in binge eating, and 25% to 47% exercise excessively to lose weight [18]. A study has also highlighted that young adults pursuing careers in nursing and medicine face an elevated risk of developing eating disorders [19].

Published studies indicate that the likelihood of eating disorders is higher in females compared to males [20, 21]. Investigations conducted in various countries have identified elevated levels of body image worries among medical students [22–24]. Among Pakistani medical students, a substantial proportion (78.8%) expressed concerns about their physical appearance, and a small percentage (5.8%) met the criteria for Body Dysmorphic Disorder [22]. As a result, it is important to undertake additional research in this specific student population to gain deeper insights into this subject.

India is increasingly recognizing the imperative to respond promptly to the pressing issue of eating disorders [25]. The prevalence of these disorders was not reported in India until the late twentieth century [26]. The recent upsurge in ED cases may be attributed to glorifying the “size zero” body type in media and the cultural sanction provided to thinness and ever-present body shaming. Consequently, abuse of laxatives, self-induced vomiting, starvation, and over-exercising manifest into a full-blown ED.

A crucial risk factor for the emergence and perpetuation of most eating disorders is determined to be body dissatisfaction. A cross-sectional study of 555 female college students in North India found negative body image to be connected with greater BMI, less self-esteem, more neuroticism, and conscientiousness among a significant number of young women [27]. Thus, it is important to recognize individual differences in personality traits as well as self-esteem while attempting to understand body image concerns and eating disorder behaviour.

Since body image is based on a social concept of ideal body image, it is essential to evaluate it in its cultural context. Despite the growing awareness of mental health issues among medical students, there is a lack of comprehensive research specifically focusing on body image concerns in this population in India. Filling this gap can provide valuable insights into the prevalence, causes, and potential solutions for such concerns. As future healthcare professionals, medical students play a pivotal role in shaping patients' health behaviors and attitudes. Understanding their own body image concerns can influence how they approach patient care, helping them develop empathy and sensitivity toward similar issues in their patients. According to our current understanding, no published Indian studies research the relationship among body image perception, perceived stress, eating disorder behaviour, and life quality in female medical students.

## Methodology

Disordered eating behaviour is defined as “a preoccupation with weight and food, crash diets, fasting, binge eating, and purging behaviours” [28].

Female medical students (n = 777) were recruited by convenience sampling. The study followed a Cross-sectional Observational Research Design.

### Tools

A socio-demographic questionnaire was developed, including age, financial situation, and family characteristics; the BMI was calculated for each individual. Other standardised tools used:

1. Body Shape Questionnaire (BSQ).

It is a self-report assessment tool for the preoccupations with body image that are typical of anorexia nervosa and bulimia nervosa [29].

(2) Body Image Quality of Life Inventory (BIQLI).

The BIQLI assesses how much an individual body image influences their life quality. Each item is answered on a 7 point likert scale by the subjects [30]. Participants score their level of agreement with 19 statements regarding how their body image impacts them on a scale of − 3 (very negative influence) to + 3 (extremely positive effect). BIQLI has an internal consistency with Cronbach's alpha 0.9.

(3) Eating Attitudes Test-26 (EAT-26).

The Eating Attitudes Test-26 (EAT-26), a 26-item scale that examines attitudes towards food and eating, was used to assess disordered eating behaviour [31]. Participants used a 6-point scale to indicate how frequently each item relates to them (0-'never,' 'rarely,' or'sometimes, ‘1’-often, ‘2’-usually, ‘3’-always’). A score of 20 or above suggests the risk of an eating disorder. Internal consistency and validity of the EAT-26 range from fair to excellent.

(4) Rosenberg Self-Esteem Scale [32] (RSES).

A 10-item Rosenberg self-esteem scale (RSES) has been recognised as the bench mark of self-esteem measurement [33]. The respondent answered one of the ten items on a Likert scale of 1–4: You can choose from “1” (“strongly disagree”), “2” (“disagree”), “3” (“agree”), and “4” (‘strongly agree’). RSES holds good construct validity and reliability [34].

### Procedure

List of all the students in a medical college was obtained and they were approached through email and whatsapp with an invitation to take part in this study. Online informed consent was taken. The survey was sent in the form of a google form link. The survey was in English language. The students were well-versed in English as the medical studies are in the same language (English).

The students were contacted three times over a period of two weeks with reminders to participate in the study. Initially there was a 50% response, 30% with the second reminder and 20% in the third reminder. All of them were given the phone number of principal investigator to seek help for mental health concerns. Those who were identified to have mental health issues were given feedback and were offered treatment.

### Human ethics and consent to participate declarations

All subjects gave their informed consent for inclusion before they participated in the study. The study was conducted in accordance with the Declaration of Helsinki, and the protocol was approved by the institutional ethics committee [IEC No. 1331]. Those who were diagnosed with mental health issues were offered treatment by the principal investigator.

### Statistical analysis

The data were analysed using SPSS software for Windows (version 23.0). Descriptive statistics using mean and standard deviation for continuous, and frequency and percentage for categorical variables. Alternatively, median and inter-quartile range was used to describe continuous variables with skewed distribution. The proportion of participants who screened positive for a risk of eating disorder were determined using the EAT-26 threshold score as described in the description of this instrument above. The data was checked for normal distribution using the Kolmogorov–Smirnov test, and appropriate non-parametric tests were applied in view of significantly skewed distribution for EAT-26, BSQ, BIQLII, and RSES scores. Bivariate analysis using appropriate inferential statistics (Mann–Whitney U test, Spearman correlation, Chi-square test) were conducted to examine the bivariate associations between different variables and eating disorder behavior. Fisher’s Exact test was applied when expected cell frequency of less than five was reported in one or more cells of the 2 × 2 contingency table. A multivariate analysis was performed using the binary logistic regression with eating disorder screen status as the dependent variable, and all factors having significant bivariate association with it entered as independent variables in the model. About 3.2% (n = 26) participants had either given improbable answers or left blank responses to one or more questions in the survey, and were excluded from the analysis. No missing value imputation was conducted. Statistical significance for all tests was set at p-value of less than 0.05.

## Results

The women-only college had a strength of 850 students including interns at the time of the study. We sent invitation to participate to all the students and interns. Out of the 850 students, 54 did not give consent. Data was excluded from 19 students who gave incomplete responses. A total of 777 female college students with average age of 20.62 (SD: 1.58) years comprise the study sample. Table 1 described the relevant socio-demographic and clinical characteristics of study participants.

**Table 1:** Socio-demographic and clinical profile of study participants (N = 777)

Study variable	Mean ± SD/ Median (IQR) or frequency (percentage)
Age (In years)	20.62 ± 1.58
Year of study:	
First professional	182 (23.4%)
Second professional	184 (23.7%)
Third professional	331 (42.6%)
Internship	80 (10.3%)
Body Mass Index (In Kg/m^2^):	21.87 (19.48–24.87)
H/o diagnosed psychiatric illness:	33 (4.2%)
H/o psychiatric treatment:	17 (2.2%)
H/o binge eating for at least 2–3 times a month in past 6 months:	186 (23.9%)
H/o self-induced vomiting for at least once a month in past 6 months:	61 (7.9%)
H/o use of laxatives or medications to control one’s weight for at least once a month in past 6 months:	36 (4.6%)
H/o exercising (> 60 min) to reduce or control one’s weight for once a day or more over past 6 months:	34 (4.4%)
H/o weight loss of 9 kg or more in past 6 months:	29 (3.7%)
EAT-26 score	6.00 (3.00–12.00)
BSQ score	29.00 (21.00–43.00)
BIQLI score	2.00 (- 3.00–11.00)
RSES score	23.00 (21.00–24.00)

SD, standard deviation; IQR, inter-quartile range; H/o, history of; EAT-26, eating attitudes test; BSQ, body shape questionnaire; BIQLI, body image quality of life inventory; RSES, rosenberg self-esteem scale

The mean and median EAT-26 score were 8.24 and 6.00 respectively, with about 7.5% (58/777) of them scoring above the threshold score of 20 and screened positive for at-risk of an eating disorder. There was significant correlation of the total EAT-26 score with the BSQ (ρ = 0.52, *p* < 0.01), BIQLI scores (ρ = – 0.13, *p* < 0.01), and RSES scores (ρ = – 0.20, *p* < 0.01) among study participants. The bivariate analysis assessed the relationship of different study variables with eating disorder behavior (see Table 2). Eating disorder behaviour was significantly more common among participants reporting binge eating for at least 2–3 times a month in past 6 months (*p* < 0.01), self-induced vomiting for at least once a month in past 6 months (*p* = 0.003), use of laxatives or medications to control one’s weight for at least once a month in past 6 months (*p* = 0.003), and weight loss of 9 kg or more in past 6 months (*p* = 0.004). Also, eating disorder status was significantly associated with BMI (*p* < 0.01), BSQ score (*p* < 0.01), BIQLI score (*p* < 0.01), and RSES score (*p* < 0.01).

**Table 2:** Bivariate association between study variables and eating disorder behavior (N = 777)

Study variable	Eating Disorder Screen Positive (n = 58) [Mean ± SD/ Median (IQR) or Frequency (percentage)]	Eating Disorder Screen Negative (n = 719) [Mean ± SD/ Median (IQR) or Frequency (percentage)]	Test statistic (*p*-value)
Age (In years)	20.62 ± 1.54	20.62 ± 1.58	^a^ 0.03 (0.97)
Body Mass Index (In Kg/m^2^)	24.57 (21.49–29.33)	21.51 (19.39–24.67)	^d^ 13,084.50 (< 0.01)*
H/o self-reported psychiatric illness:	5 (8.6%)	28 (3.9%)	^c^ 2.94 (0.09)
H/o receiving psychiatric treatment:	2 (3.4%)	15 (2.1%)	^c^ 0.46 (0.36)
H/o binge eating for at least 2–3 times a month in past 6 months:	29 (50.0%)	157 (21.8%)	^b^ 23.38 (< 0.01)*
H/o self-induced vomiting for at least once a month in past 6 months:	11 (19.0%)	50 (7.0%)	^c^ 10.73 (0.003)*
H/o use of laxatives or medications to control one’s weight for at least once a month in past 6 months:	8 (13.8%)	28 (3.9%)	^c^ 11.90 (0.003)*
H/o exercising (> 60 min) to reduce or control one’s weight for once a day or more over past 6 months:	5 (8.6%)	29 (4.0%)	^c^ 2. 96 (0.10)
H/o weight loss of 9 kg or more in past 6 months:	7 (12.1%)	22 (3.1%)	^c^ 12.12 (0.004)*
BSQ score	55.00 (41.75–74.00)	28.00 (21.00–41.00)	^d^ 6260.50 (< 0.01)*
BIQLI score	- 5.50 (- 18.50–6.25)	2.00 (- 2.00–11.00)	^d^ 13,049.00 (< 0.01)*
RSES score	21.00 (20.00–23.00)	23.00 (21.00–24.00)	^d^ 14,953.00 (< 0.01)*

SD, standard deviation; IQR, inter-quartile range; H/o, history of; BSQ, body shape questionnaire; BIQLI, body image quality of life inventory; RSES, rosenberg self-esteem scale; ^a^Independent t-test; ^b^Chi-square test; ^c^Fishers Exact test; ^d^Mann Whitney U-test; *p-value < 0.05

The Hosmer and Lemeshow test (for goodness of fit) was not significant (χ^2^ = 5.56, *p* = 0.69), implying that the model predicted was sound. The overall logistic regression model was statistically significant (χ^2^ = 110.34; *p* < 0.001) and explained 32.1% of the total variance observed in the eating disorder status (Table 3). The model had an overall accuracy of 92.5% in correctly classifying the participants as with and without eating disorder behavior as per the EAT-26 cut-off score value of more than 20 for screening them. The participants with history of weight loss of more than 9 kg in past six months [Odds ratio (OR): 3.09; 95% confidence interval (CI): 1.00–9.56], and a higher BSQ scores indicative of greater degree of body shape concerns (OR 1.07; 95% CI 1.05–1.09) were more likely to screen positive for eating disorder behavior on EAT-26 item score.

**Table 3:** Results of binary logistic regression analysis of variables associated with eating disorder (N = 777)

Study variable	OR	95% CI	p-value
Body Mass Index (In Kg/m^2^)	1.00	1.00–1.00	0.52
H/o binge eating for at least 2–3 times a month in past 6 months:	1.61	0.82–3.15	0.15
H/o self-induced vomiting for at least once a month in past 6 months:	0.78	0.27–2.26	0.64
H/o use of laxatives or medications to control one’s weight for at least once a month in past 6 months:	1.58	0.48–5.21	0.44
H/o weight loss of 9 kg or more in past 6 months:	3.09	1.00–9.56	0.04*
BSQ score	1.07	1.05–1.09	< 0.01*
BIQLI score	0.99	0.98–1.01	0.95
RSES score	0.93	0.87–1.00	0.06

*p < 0.05. OR, Odds ratio; CI, Confidence interval; BSQ, body shape questionnaire; BIQLI, body image quality of life inventory; RSES, rosenberg self-esteem scale

Cox-Snell R^2^ = 0.132; Nagelkerke R^2^ = 0.321

## Discussion

There have been studies worldwide on the prevalence of eating disorders but limited studies have been done in India. The prevalence of ED in India ranged from 4 to 45.4% [35]. The prevalence percentage of eating disorder vulnerability observed in medical students through a meta-analysis from 9 countries including India was greater (10.4%) [36] than the rate documented in the general population, which is approximately 5% [37]. Our study assessed body image perception, perceived stress, eating disorder behaviour, and quality of life among female medical students in India among the age groups ranging from 19 to 21 years. The average body mass index (BMI) was 21.87, similar to that reported in RisC & NCD [38], which means BMI was more than 20•7 in women in India. 

In our study sample, 7.5% screened positive for an eating disorder behaviour comparable to a study of 1600 students that found 10.6% of females scored high in EAT-26 [39]. Subsyndromal forms of ED behaviour are detected in India [40]. The range of estimates regarding the susceptibility to eating disorders among medical students varies widely in different research, spanning from 2 to 30%. [41, 42] Previous researches have documented elevated risks and occurrences of eating disorders in medical students due to the demanding academic obligations, substantial workload, youthfulness, higher BMI, concerns about body and self-image, as well as exposure to illnesses and mortality during their medical education [36, 43, 44].

Female medical students seemed to face a heightened risk of eating disorders, displaying a prevalence of 13.7% (95% CI 6.6–20.7%), in contrast to the prevalence of eating disorders among their medical student counterparts [36]. Considering the prevalence of eating disorder risk and the exclusive prevalence rate for eating disorder risk among females, it becomes evident that there exists a distinction between males and females in terms of eating disorders. Historically, eating disorders have predominantly manifested in young women [45]. This may be attributed to young females possessing distinct perspectives regarding food and body weight, striving to attain and uphold a slender physique. Significance is placed on being thin as a necessary factor for self-esteem [46, 47]. However, eating disorders may be less represented in males compared to females. Social stigma and gender stereotypes often discourage males from reporting or seeking help for eating disorders [48].

In our sample, eating disorder behaviour was significantly more common among participants with reports of binge eating, self-induced vomiting, availing laxatives or medications to reduce one’s weight, and weight loss of 9 kg or more. Those with symptoms of anorexia had a significant likelihood of being diagnosed with an ED later on [49].

A significant correlation was seen between eating disorder behaviour and perceived body shape, body image, quality of life, and self-esteem among our study participants. This follows previous findings on body image and eating disorders which found that eating attitudes were significantly associated with body image concerns [50].

We also found eating disorder status was significantly associated with BMI, perceived body shape, quality of life, and self-esteem in our study participants. Females tend to choose a more petite figure as compared to their present body shape as the ideal body shape as society tends to consider looks more critical in females [3]. Parents, peer influence, media, and cultural invasion further reinforce this [51].

Previous research has established a link between one's own opinion of their physical appearance, their body mass index, and their sense of self-worth [27]. This highlights the tendency in females to pursue a thin body image ideal, and in the absence of achieving it, losing their self-esteem and have a lower quality of life.

Researchers hypothesize that idealistic cultural norms for the female body are impractical to realize fully, and females who completely internalize these, connect the achievement of these norms with their identity [52]. Consequently, there is a feeling of shame when they do not measure up. This results in lowered self-esteem [50]. It has also been observed that when females with low self-esteem do not achieve a societal flawless body shape or misinterpret their body shape, they are susceptible to environmental influences to conform to the cultural body ideal over time [53]. Reduced self-esteem also predicts behaviour like dieting and exercise, increasing the individual’s proneness to develop eating disorder behaviours [54]. These findings suggest a two-way connection uniting perceived body shape and self-esteem.

A greater degree of body shape concerns was likely to result in eating disorders. Researchers attribute this to a “thin-ideal” belief among females, which leads to dissatisfaction with their body shape leading to normal or underweight females perceiving themselves as overweight [55, 56].

Even though medical students have access to biomedical expertise and bear significant responsibility in diagnosing and treating eating disorders, they often postpone seeking assistance and treatment when they themselves are affected by these disorders [57]. This reluctance stems from concerns about facing stigma or being scrutinized regarding their suitability for a career in medical practice [58].

### Strengths and limitations

One advantage of our research is that it’s a novel attempt to investigate the interconnection between perceived body shape, BMI, body image quality, and self-esteem among young female Indian medical students. We chose the medical college group for our study because this issue is more prevalent among young adult women than older women. The relatively large sample size is a major strength of our study. However, our research has a few constraints.

Body image is a multifaceted and intricate concept. BSQ may have only partially captured the full range of body image concerns. Second, because this is a cross-sectional study, It’s challenging to establish a cause-and-effect link between perceived body shape, self-esteem, and body image quality of life, as this relationship can be bidirectional. Another limitation is a selection and self-report bias. The weight was self-reported in the study. Data was collected through a self-reported questionnaire which may cause reporting bias. Since this research is conducted on female medical college students, generalizability may be an issue. However, according to clinical and scientific data, this community is at a vital peril for body image issues and consequent eating disorders [36].

A cohort study with one-to-one interviews with the study population may overcome some limitations. Future studies could look at perfectionism as a possible related personality trait.

## Conclusion

The present study reports that elevated BMI and a greater degree of body shape concern lead to a lower body image, quality of life, and self-esteem and an increased risk for developing eating disorder behaviour in female medical students in India. This is of clinical implication to female medical students and healthcare professionals to engage early in primary and secondary prevention of eating pathologies. Increasing awareness of these facts among female students can help identify at-risk students and help the students seek timely medical support.

## Data Availability

The datasets used and analysed during the current study are available from the corresponding author on reasonable request.
